# Disentangling microstructure and environmental conditions in high‐altitude Andean microbialite systems (Catamarca, Argentine Puna)

**DOI:** 10.1111/1758-2229.13128

**Published:** 2022-10-03

**Authors:** Micaela Della Vedova, Patricio G. Villafañe, Carlos Cónsole‐Gonella, Anelize Bahniuk Rumbelsperger, Leonardo Fadel Cury, Luis R. Horta, María E. Farías

**Affiliations:** ^1^ Laboratorio de Investigaciones Microbiológicas en Lagunas Andinas (LIMLA) Planta Piloto de Procesos Industriales Microbiológicos (PROIMI), CONICET San Miguel de Tucumán Tucumán Argentina; ^2^ Instituto Superior de Correlación Geológica (INSUGEO), CONICET‐ UNT Yerba Buena Tucumán Argentina; ^3^ LAMIR Institute Universidad Federal do Paraná (UFPR) Curitiba Paraná Brazil

## Abstract

The study of microbialites development is a key tool to understand environmental pathways during deposition. We provide a detailed analysis of modern Central Andean microbialites from high‐altitude lakes. The stratigraphic record of Turquesa Lake shows a significant short‐term recolonization by microbialite‐producing microorganisms during environmental stress. Far from a crisis paradigm, the coasts and paleocoasts of Turquesa lake exhibit three microbialitic buildups formed along different stages, providing a good study case of biological resilience of these systems in harsh environments. The MI and MII microbialite buildups occupied two paleocoasts. Both are composed of oncoids with micritic to microsparitic textures. Morphological, textural and mineralogical similarities between the two buildups suggest that they were formed at different times, but under very similar environmental conditions. The microorganisms that produced the microbialitic buildup MIII are currently colonizing the coast of this lake. The previous oncoid morphology change to a parallel micritic–spartic lamination. This remarkable changes in the microstructure can be explained by an important environmental change caused by the isolation of the Peinado Lake, and a subsequently microorganism adaptation. This microbialite structures can be proposed as an interesting modern analogue for environmental changes along the geological record.

## INTRODUCTION

Microbialites were defined as organo‐sedimentary structures formed by the interaction between benthic microbial communities and detrital and/or chemical sediments (Burne & Moore, 1987; Riding, 2008). These structures can be developed over hundreds or thousands of years (Gomez et al., 2014; Jahnert & Collins, 2012), and its internal morphology is the result of the interaction between intrinsic factors (microbialite‐producing microorganisms) and extrinsic factors (environmental parameters) (Dupraz et al., 2006). If during their growth the intrinsic or extrinsic factors change, the internal structure will reflect those variations. This establishes a direct relationship between the structure in all its scales, and factors such as the distribution of microbial communities, energy of the system, drain and water supply, sedimentation rate and mineral precipitation (Dupraz et al., 2006; Golubic, 1976).

Since late Pleistocene until present, the Central Andean region had experience climatic fluctuations during short periods of time (Abbott et al., 2003; Alonso et al., 2006; Strecker et al., 2007). The sedimentary record of Central Andes endorheic lacustrine systems is an excellent indicator of these climatic fluctuations (Abbot et al., 1997; Grosjean, 1994; Valero‐Garcés et al., 2000). In addition to this, some of these water bodies exhibit very important microbialitic deposits (Farías et al., 2020) that were developed during the Pleistocene/Holocene, providing an excellent tool for climate change analysis.

**FIGURE 1:**
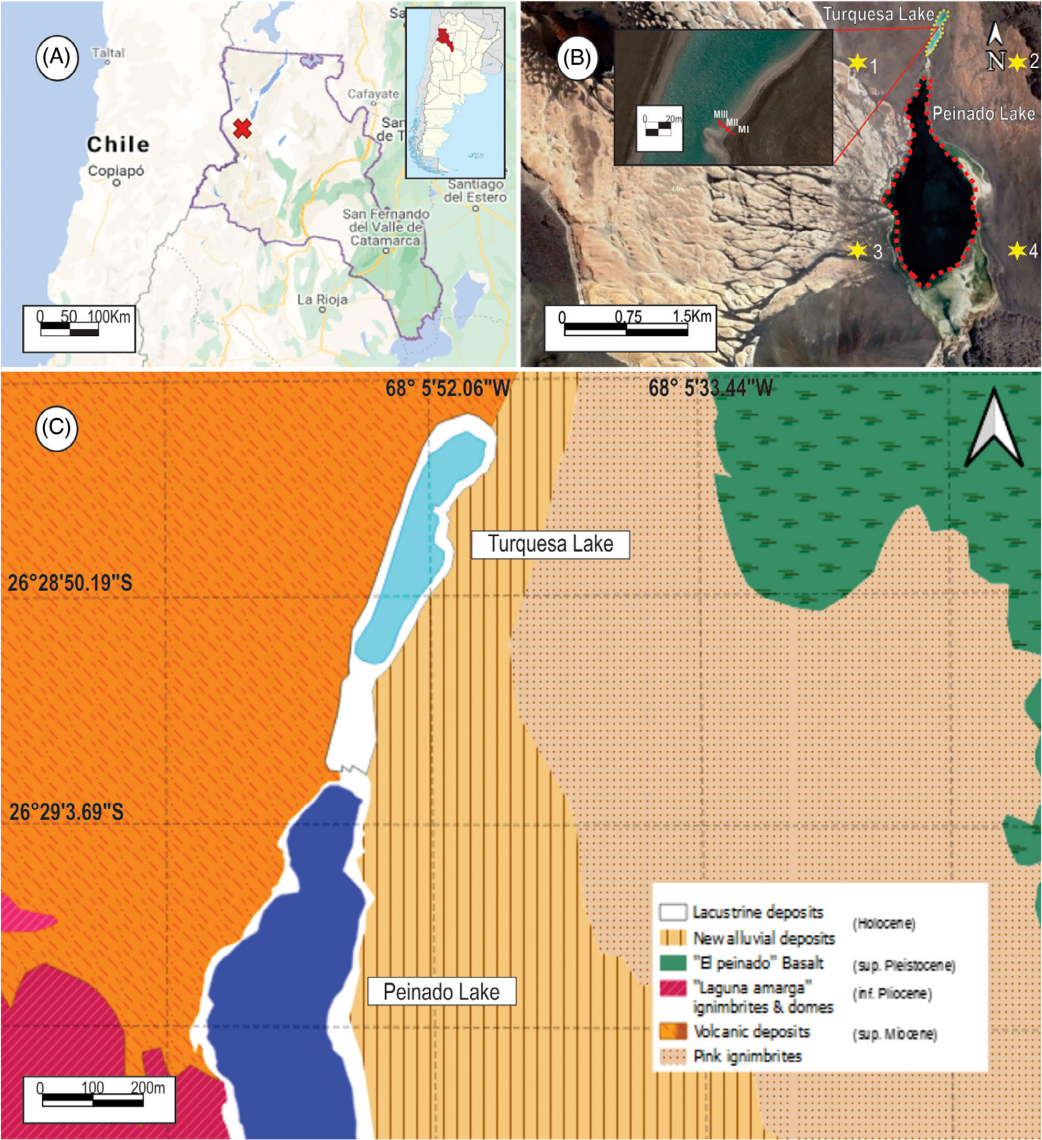
(A) Localization of the study area, in Catamarca, Argentina. (B) Satellite image and delimitation of the Turquesa and Peinado lakes. Also, an ampliation of the Turquesa Lake with the position of the zones where the samples were taken can be seen in the superior left part, being MIII the closer to the lake an MI the farthest. (*) 1: 26°29′10.08″ S, 68°6′29.37″ O; 2: 26°29′10.08″, 68°5′8.95″ O; 3: 26°30′31.32″ S, 68°6′29.37″ O; 4: 26°30′31.32″ S, 68°5′8.95″ O. (C) Geological map of the surrounded areas of Turquesa lake

**FIGURE 2:**
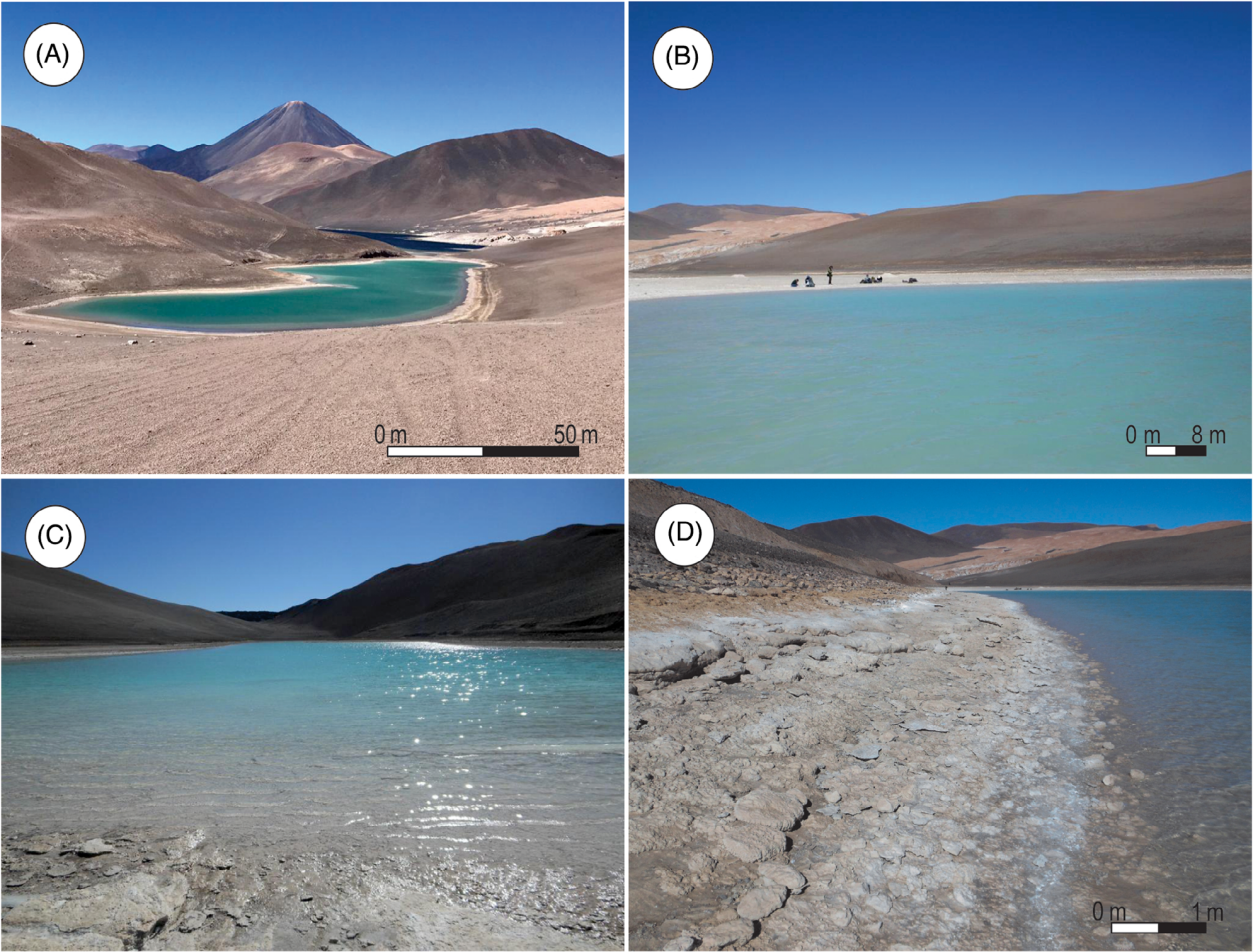
(A) North–South view of the Turquesa Lake. (B) West coast of the Turquesa Lake. (C) South–North view of the Turquesa Lake and the Peinado Lake behind. (D) Detail picture of the coast of the Turquesa Lake and microbialitic levels

Turquesa Lake (Figures 1 and 2) is an endorheic water body in the Central Andean region in the Argentinean Puna (Catamarca), just 250 m north of Peinado Lake. Previous literature suggests that these lakes have been under a hydrological crisis during the last few decades (Valero‐Garcés et al., 2000, 2001). This crisis led to rises and falls in the water level, causing episodes of connection and disconnection between Turquesa Lake (Figure 2) and Peinado Lake, affecting the physicochemical conditions of the environment (Villafañe, Cónsole‐Gonella, et al., 2021).

**FIGURE 3:**
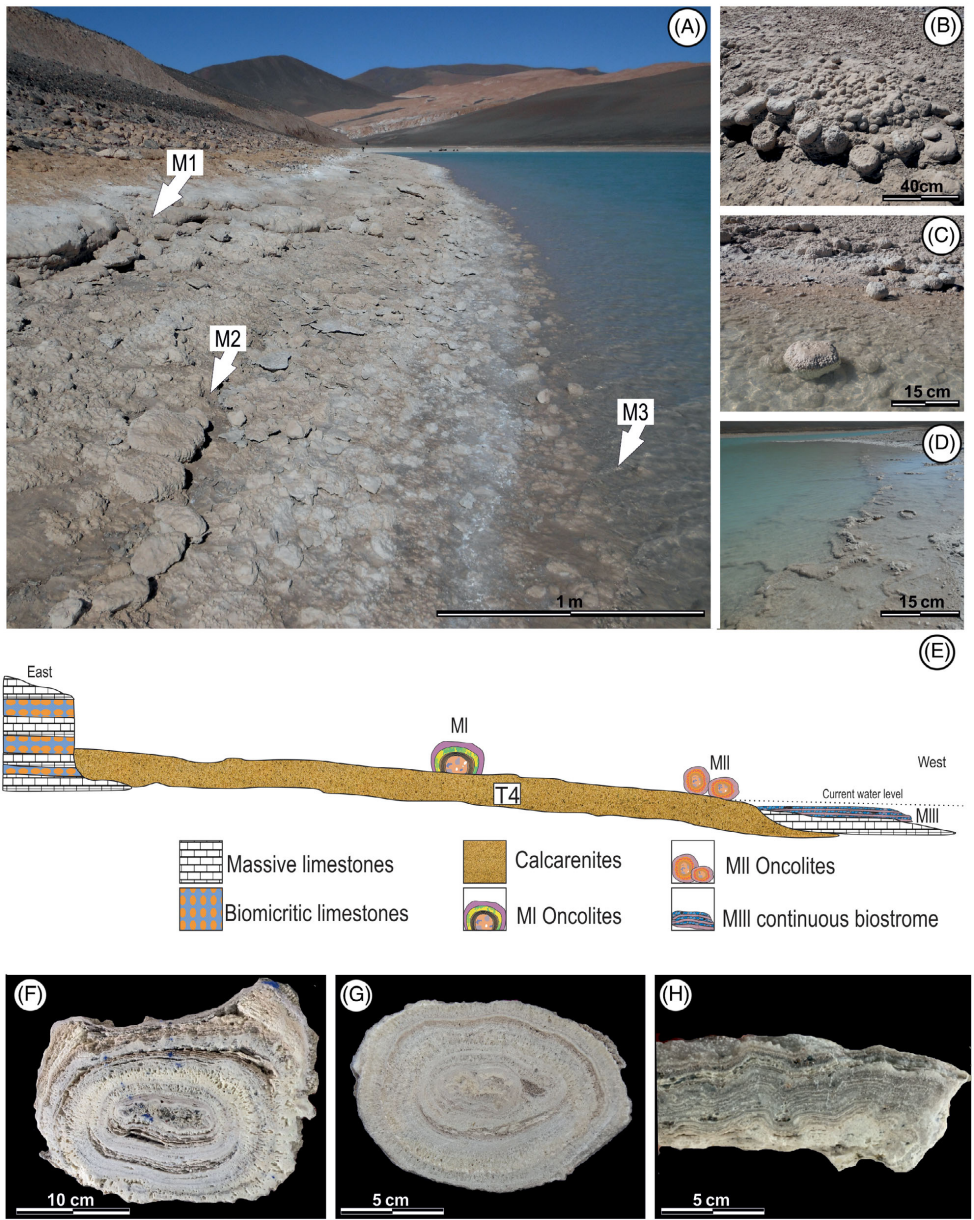
(A) Field view of the three microbialitic levels. (B, C) Oncolites of levels MI and MII. (D) Level MII. (E) Profiled view of the Turquesa lake and its three microbialitic levels. *Source*: Modified from Villafañe, Cónsole‐Gonella, et al., 2021; Villafañe, Lencina, et al., 2021 . (F–H) Inside view of the mesotructures of MI MII and MIII, respectively

In the stratigraphic record of Turquesa Lake, Villafañe, Cónsole‐Gonella, et al. (2021) have identified three Holocene microbialitic buildups on its coast and paleocoast (MI, MII and MIII) (Figure 3A). Two of them (MI and MII) were observed as paleocoasts, with individual structures that have a very similar external morphology and with oncoid type internal structures (up to 20 cm and 12 cm diameters, respectively). While the third one is currently below the water level, up to 50 cm depth, presenting a biostrome‐type morphology with an internal structure characterized by the alternation of parallel laminae. Although Villafañe, Cónsole‐Gonella, et al. (2021) suggest that each microbialitic buildup represents a different environmental stage of the lake; their hypothesis has not yet been confirmed by a detailed microstructural study.

This contribution proposes a detailed analysis of the microstructure of the microbialitic buildups in the stratigraphic record of Turquesa Lake, seeking to identify the degree of influence of the previously mentioned episodes of connection and disconnection between the lakes during the microbial colonization of this water body. Knowing the dominant building blocks for each microstructure and identifying the growth mechanisms that gave rise to them will allow us to discuss the resilience of microbialite‐producing microorganisms to climatic/environmental changes, offering a key approach to understand analogue processes throughout the Earth history.

## EXPERIMENTAL PROCEDURES

To start, a delimitation of the study area and a detailed survey of the microbialitic buildups in Turquesa Lake was conducted through. Based on this, for each level, we recognize a predominant macroscopic shape and structure, taking this in to consideration that we proceeded to a systematic sampling of the microbialitic buildups. One sample for each microbialitic buildup, has been chosen, being these the most representatives for the shape and structure of each level. The approximately position on the sampling location was S 26°28′44.63″ S, N 68°5′51.46″ O, on the East margin of the Turquesa Lake. MI sample was take approximately 6.5 m from the shoreline, MII 2.5 m from MI and finally, MIII was taken under the water surface in the shore line (4 m distance to MII). All the microbialitic buildups were elevated less than 1 m from each other and from the shoreline.

Data taken in the lake such as pH, total dissolved solids, conductivity and water temperature were taken by Villafañe, Cónsole‐Gonella, et al. (2021).

Microstructure (microscopic fabrics observed under petrographic analysis) (Shapiro, 2000; Vennin et al., 2015) was analysed at the laboratory of the Instituto Superior de Correlación Geológica (INSUGEO, CONICET‐UNT) through the use of polish slabs and thin sections. For the sample preparation, in the case of MI and MII (oncolite shape) points at the nucleus, the middle and the external part were chosen, for MIII we chose just one section as it is mostly homogeneous in its morphology. Then, these sections have to be cut with a specific tool due to their hardness, put in a thin glass section and abrade to get to a thickness that is optimum (0.03 mm) for the light of the petrographic microscope to go through it. With this method, we were able to determinate parameters like lamination types, stacking, lateral, and vertical continuity of lamination, growth dynamics and hiatuses. All the images in this work were taken with crosspolars.

Lamination was analysed by describing factors such as composition, lateral continuity, thicknesses, geometrical arrangement, among others. Carbonates description has been made using Dunham (1962). The percentage of porosity was estimated visually by comparation with quantification charts like (Baccelle & Bosellini, 1965) and it has been classified according to Choquette and Pray (1970) proposal.

In addition to this, a qualitative and semi‐quantitative determination of the minerals that make up the microbialitic buildups was performed using x‐ray diffractometry in LAMIR Institute, Federal University of Parana (Curitiba, Brazil). For this, representative samples of each microbialitic buildup were pulverized and homogenized in its whole, by using agate mortar (total rock). Subsequently, a Panalytical brand x‐ray diffractometer (EMPYREAN model) with an X'Celerator detector and copper tube was used to carry out the study. Mineral fractions were analysed with Malvern PANalytical Empyrean, with X'Celeratore detector and Cu tube was applying scan rate of 0.5°/min, under a voltage of 40 kV and current of 30 mA. Finally, the interpretation of the results has been done with the X'Pert Highscore Plus Software (PANalytycal) with PDF‐2 data bank. To complete this work, a radiocarbon dating was carried out in LATYR (laboratory of radiocarbon), in La Plata, Buenos Aires, Argentina. For this we, chose a point in the most external layer of MI (the oldest microbialitic buildup according to stratigraphy), and taking in to consideration that MIII level (currently forming under water) is the most resent one. For this, the correction factor was: d^13^C (estimated): −10 ± 2‰ and the multiplication Factor of the error was (K) = 1 (The calibration for the south hemisphere SHCal20. 14c Hogg et al., 2020: Radiocarbon 62). With this process, we get an age of 14C 11.830 ± 170 years AP.

## GEOLOGICAL SETTING AND CLIMATE

The Argentinean Puna is a morphotectonic unit of the Central Andes, elevated approximately 3700 m above sea level (Alonso & Rojas, 2020). This region is characterized by a steep local relief, caused by contractional ‘basins and ridges’ and volcanoes (Kraemer et al., 1999) (Figure 1). In addition to this, an arid to semi‐arid climate with endorheic watersheds allows the formation of evaporation environments such as lakes, salt plains and wetlands (Alonso et al., 2006; Alonso & Rojas, 2020; Jordan & Mpodozis, 2006).

Throughout its extension, the Puna region presents a diversity of basins composed by continental‐evaporitic Pleistocene deposits, in addition to volcanic and clastic deposits (Alonso et al., 1991; Alonso & Rojas, 2020; Strecker et al., 2007) (Figure 1). One of these is the El Peinado basin, which is located south of the Antofalla Salt flat (Catamarca) and San Buenaventura Mountain range. This basin belongs to the active Ojo del Salado volcanic region, in the Central Andean Volcanic Province (Valero‐Garcés et al., 2000), and was formed by tectonic events and volcanic activity during the Plio‐Pleistocene (Valero‐Garcés et al., 2001).

Inside El Peinado basin, a depression which two water bodies separated by only 250 m stands out (Figure 2A). To the north: Turquesa Lake (26°39′14″ S, 68°10′42″ O) (Figure 1), on which this study focuses, presenting a 0.1 km^2^ surface and 6 m average deep. Parameters were measured in the field by Villafañe, Cónsole‐Gonella, et al. (2021) and they mention for this lake a pH of 7.58, a conductivity above 100 mS/cm and a concentration of total dissolved solid above 60 mg/L; while the average temperature is around 9.15°C. To the south is the Peinado Lake, with a surface of 1.6 km^2^ and around 8 m deep. Measurements by the same authors in this water body indicates a pH of 7.91, a conductivity of 65.20 mS/cm, a concentration of total dissolved solids of 39.10 mg/L and average temperature of 9.15°C. Furthermore, in the south of this lake three zones of hydrothermal supply stands out, with temperatures reaching up 33.13°C (Valero‐Garcés et al., 2001; Villafañe, Cónsole‐Gonella, et al., 2021). Measurements such as pH, total dissolved solids, conductivity and water temperature, taken by Villafañe, Cónsole‐Gonella, et al. (2021) on the water of Turquesa Lake were sample at the point: N 26°28′56.6″, W 68°05′ 56.8″.

Around Turquesa and Peinado lakes, four paleoscoast were recognized (Figure 2D). The oldest one (TI, TII and TIII) presents a silicoclastic composition with conglomerates and sandstones (Valero‐Garcés et al., 2001). The following paleocoast (TIV) shows a carbonatic composition and in Turquesa Lake two paleocoast with microbialitic buildups were recognized (MI y MII) and associated with sedimentary structures such as ripple marks (Villafañe, Cónsole‐Gonella, et al., 2021; Villafañe, Lencina, et al., 2021). Finally, a younger deposit was developed below the currently water level of Turquesa Lake, in which the microbialitic buildup MIII was observed (Figure 2D) (Villafañe, Cónsole‐Gonella, et al., 2021; Villafañe, Lencina, et al., 2021). The external layer of the microbialitic buildup MI (the oldest) was dated with radiocarbon (see Experimental and Procedures) and has an average age of 14C 11.830 ± 170 yeas BP. This means that MI microbialite build up is Upper Pleistocene in age and that MII and MIII are younger due to their stratigraphic position.

In recent years, microbial ecosystems of the Argentinean Puna have attracted considerable attention due to the extreme climatic/environmental conditions under which they have been developed. The temperature of this region can vary daily in a range that goes from 10 to 20°C in summer (south hemisphere season) and 10–40°C in winter (south hemisphere season), meaning that the temperature difference between day and night is larger in the last‐mentioned season. In addition, due to its latitude and other factors, the Andean valleys are one of the most irradiated zones in the word (with a monthly average reaching 6.6 k Whm^−2^ d^−1^). Also, conditions such as high salinity (125 mS), low oxygen pressure, constant volcanic eruptions, arsenic content, high thermal fluctuation, and high aridity, added to other environmental factors, make this region very harsh for life and for ecosystems prosperity (Farías et al., 2011, 2013, 2014; Saona et al., 2020; Villafañe, Cónsole‐Gonella, et al., 2021; Villafañe, Lencina, et al., 2021).

From the late Pleistocene to the present, the Puna region underwent varied climatic fluctuations in short periods of time caused by orbital eccentricity, glaciations/deglaciations, El Niño Southern Oscillation (ENSO), among other climatological factors (Abbott et al., 2003; Alonso et al., 2006; Strecker et al., 2007). Due to the complex topography and internal drainage networks, climatic fluctuations are regionally controlled (Alonso et al., 2006; Strecker et al., 2007) and are well recorded in the sedimentary sequence of lakes and lagoons (Abbott et al., 2003; Alonso et al., 2006).

El Peinado basin is not the exception, and in addition to all the parameters mention before, it has been in a constant hydrological crisis during the last decades (Valero‐Garcés et al., 2000, 2001). Therefore, both Turquesa and Peinado lakes have being in desiccation due to climate changes, being this the reason why the lakes are separated at the present. As a consequence of the isolation of the Turquesa Lake, the hydrothermal supply coming in the past from Peinado Lake to Turquesa Lake is no longer happening, changing many of the parameters in the lake (Villafañe, Cónsole‐Gonella, et al., 2021).

## RESULTS

### Microstructures of microbialitic buildup MI

**FIGURE 4:**
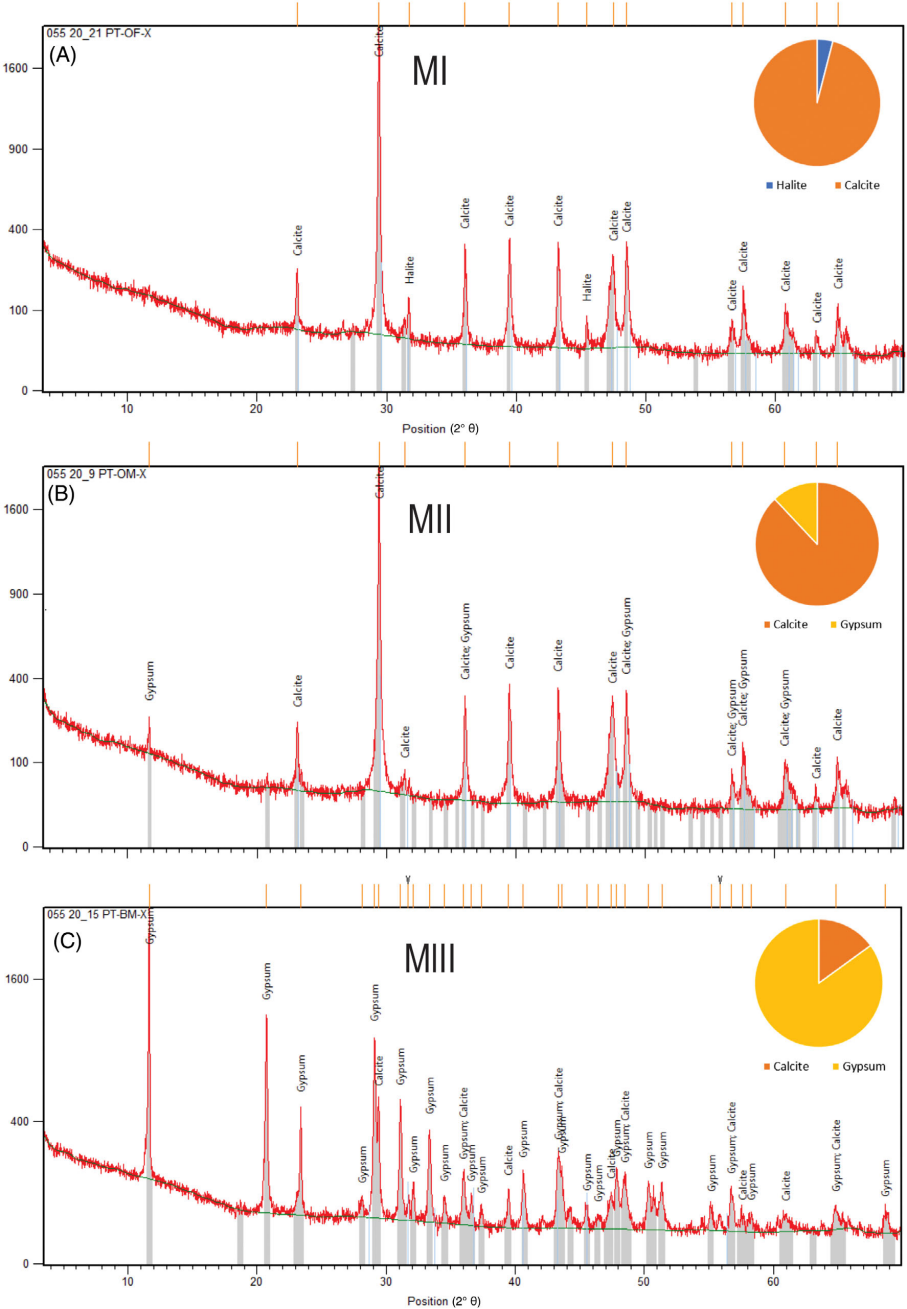
(A) X‐ray diffraction (XRD) results from microbialitic level MI, with percentages of calcite (96%) and halite 4%. (B) XRD results from MII with 88% of calcite and 12% of gypsum. (C) XRD results from MIII with a clear gypsum predominance of 85% and a 15% of calcite

MI is formed by oncolites, up to 20 cm wide and 9 cm tall, which exhibit a concentric internal structure around a nucleus (Figure 3F). Based on its textural and lithological variations, it is possible to distinguish four microstructures: (i) *Nucleus zondiame*, (ii) c*ontinuous laminated zones*, (iii) *clotted laminated zones* and (iv) *non‐laminated zones* (Table 1). The x‐ray diffractometry indicates a clear calcite predomination (micritic/microsparitic) about 96% according to the DRX software. While in less quantity, 4%, the presence of halite has been determined (Figure 4A).

**TABLE 1:** Main characteristics observed in the three microbialitic buildups from Turquesa Lake: morphology, composition determinate by the use of thin cuts, microstructures, porosity percentages and type and x‐ray diffraction (XRD) compositions (taking in to consideration the total of the sample and not each zone)

	Microbialitic buildup	Morphology	Observed composition (microscope)	Microstructures	Porosity type	Porosity % (visually estimated)	XRD composition
Water level ⇩	MI	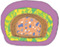 Oncolite	‐Micritic ‐Microsparitic ‐Igneous clasts	Nucleus zone	‐Intergranular ‐Cavern ‐Vug	16%–32%	Calcite: 96% Halite: 4%
				Continuous laminated zone	‐Fenestral ‐Canal ‐Fracture ‐Intergranular	8%–10% (16%–30%)	
				Clotted laminated zone	‐Fenestral ‐Intergranular	16%–32%	
				Non‐laminated zone	‐Canal ‐Fracture ‐Intergranular	16%–37%	
Current water level	MII	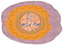 Oncolite	‐Micritic ‐Microsparitic ‐Igneous clasts	Nucleus zone	‐Intergranular ‐Cavern ‐Vug	16%–32%	Calcite 88% Gypsum 12%
				Continuous laminated zone	‐Fenestral ‐Cavern ‐Vug ‐Intergranular	16%–30%	
				Domed laminated zone	‐Intergranular ‐Cavern	16%–32%	
				Non‐laminated zone	‐Cavern ‐Intergranular	18%–25%	
	MIII	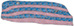 Continuous Biostrome	‐Micritic ‐Sparitic ‐Bioclasts	Intercalation of continuous and sinuous laminae	‐Fenestral ‐Intergranular ‐Intercrystal ‐Intragranular	20%	Gypsum 85% Calcite 15%

**FIGURE 5:**
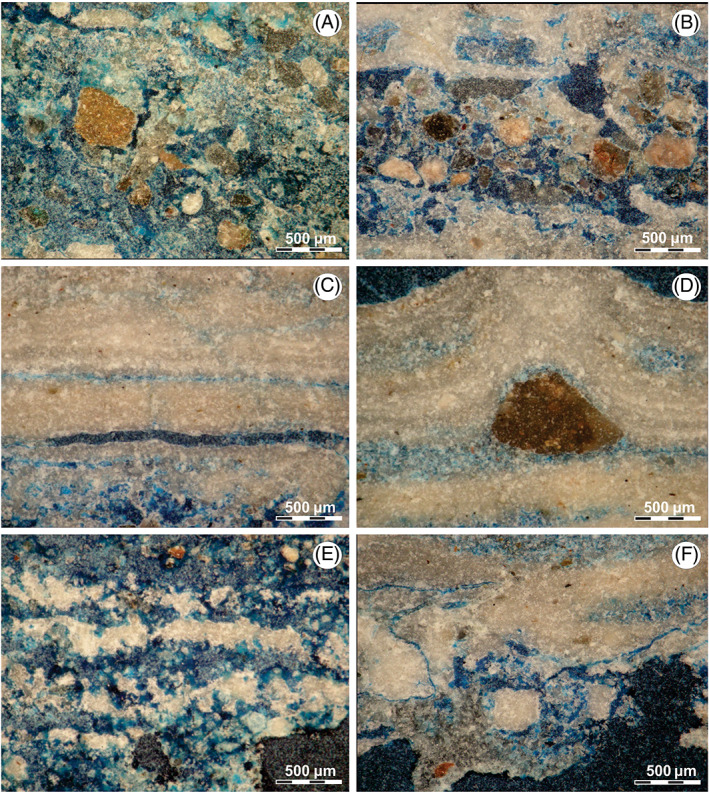
Petrographic microscope images with crosspolars: MI microstructures. (A, B) Nucleus zone of (C) laminated zone, with the presence of fenestral pore type and intercalation of dark micritic laminae with a microspartitic laminae. (D) Laminated zone with micritic intraclast interrupting the lamination. (E) Clotted laminated zones. (F) Non‐laminated zone

*Nucleus zone*: The nucleus presents igneous clasts (0.5–0.25 mm diameter) with erosive shapes and micritic clasts (up to 0.5 mm diameter). All immersed in a micritic/microsparitic matrix, conforming a mudstone/wackestone texture (Dunham, 1962) (Figure 5A,B).

The porosity percentage goes from 16% to 32%. Pores can be mainly classified as intergranular, and in a lesser extent like cavern and vug type (Choquette & Pray, 1970).

*Continuous laminated zones*: In these zones, it is possible to observe the alternation of dense and continuous laminae of micritic and microsparitic composition. Micritic laminae go from 0.25 mm from up to 0.5 mm, and microsparitic laminae are less than 0.25 mm (Figure 5C,D). The edges of the lamination are well defined on the bottom and slightly cracked on the top side (Figure 5C). In some cases, the presences of micritic clasts (up to 0.5 mm) inside the microsparitic laminae were observed, modifying the geometry of the adjacent lamination by increasing its thickness (Figure 5D).

Porosity goes from 8% to 10% (16%–30% when there is fracture evidence). Pores can be classified as fenestral, canal, fracture and intergranular type (Choquette & Pray, 1970) (Figure 5C,D).

*Clotted laminated zones*: These zones present an alternation of discontinuous clotted laminae of micritic composition, with continuous massive laminae of micritic/microsparitic composition. Micritic laminae present thicknesses up to 0.25 mm, while the thickness of micritic/microsparitic laminae goes up to 0.5 mm (Figure 5E). In some cases, the discontinuous micritic nodule that forms the clotted texture is aligned with the lamination and interleaved with the microsparitic laminae in this type of microstructure porosity goes from 16% to 32%. Pores can be classified as fenestral and intergranular type (Choquette & Pray, 1970) (Figure 5E).

*Non‐laminated zones*: The absence of lamination is characteristic of these zones. A mudstone/wackestone texture (Dunham, 1962) is observed, composed of igneous and micritic clasts immersed in a micritic matrix (Figure 5F). Igneous clasts have anhedral morphologies and diameters up to 0.25 mm, while micritic clasts have more rounded morphologies and diameters up to 0.5 mm.

Porosity occupies between 16% and 37% of the surface of thin cuts. Pore types can be classified as canal, fracture and intergranular (Choquette & Pray, 1970) (Figure 5F).

### Microstructures of microbialitic buildup MII

MII is formed by oncolites (up to 12 cm wide and 5 cm tall), which exhibit a concentric internal structure around a nucleus, very similar to MI (Figure 3G). Based on its textural and lithological variations is possible to distinguish four microstructures: (i) *nucleus zone*, (ii) c*ontinuous laminated zones*, (iii) *dome laminated zones* and (iv) *non‐laminated zones* (Table 1). The x‐ray diffractometry (Figure 4B) indicates for microbialites in MII a clear calcite (micritic/microsparitic) predomination (88%). While in a less quantity, the presence of gypsum was determinate (12%).

**FIGURE 6:**
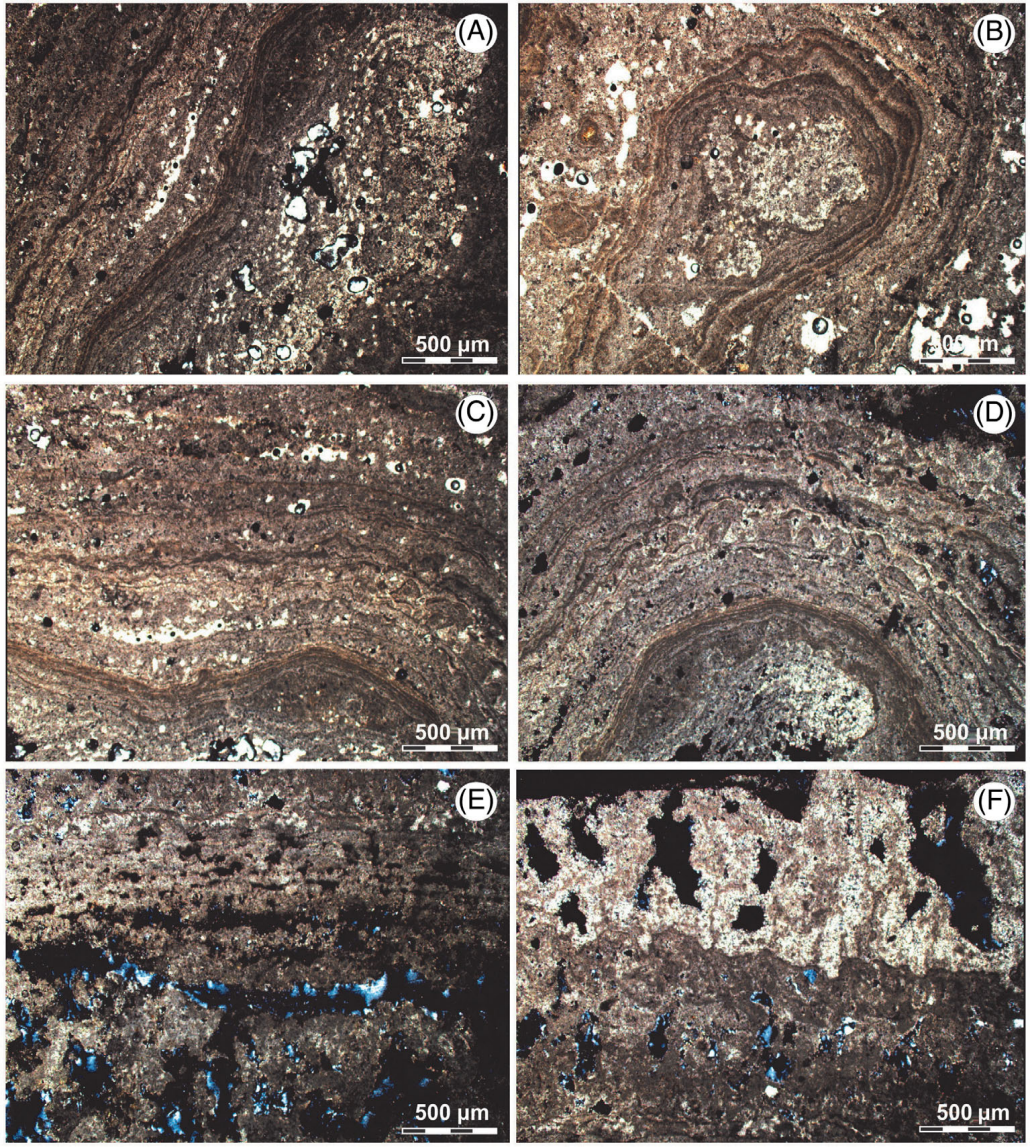
Petrographic microscope images with crosspolars: MII microstructures. (A, B) The nucleus zones continued by the laminated zones. (C, D) Domed laminated zones. (E, F) Non‐laminated zones, with crystallization in the pore space

*Nucleus zone*: The nucleus presents igneous clasts (up to 0.25 mm diameter) with rounded shapes and micritic clasts (up to 0.5 mm diameter). All these clasts are immersed in a micritic/microsparitic matrix conforming a mudstone/wackestone texture (Dunham, 1962) (Figure 6A,B).

Porosity ranges between 16% and 32%. Pores can be classified as intergranular, cavern and vug type (Choquette & Pray, 1970).

*Continuous laminated zone*: These zones present an intercalation of micritic gently wavy lamination with micritic/microsparitic laminae (Figure 6C). Micritic laminae show thicknesses of up to 0.01 mm, while micritic/microsparitic laminae can reach 0.1 mm thickness. The edges of the lamination are well defined in the bottom part and slightly cracked on the top. Sporadically intercalation of sparitic laminae (less than 0.01 mm) has also been observed (Figure 6C).

Porosity occupies between 16% and 30% of the surface of thin cuts. Pores can be classified as fenestral, cavern, vug and intergranular type (Choquette & Pray, 1970).

*Domed laminated zones*: In these zones highlight domic structures with asymmetrical morphologies inclined towards one of its flanks. The domes are composed by the alternation of micritic and micritic/microsparitic lamination, both with less than 0.01 mm thick (Figure 6D). The lamination is interrupted laterally and presents cracks that are filled with microsparitic. Sparitic lamination is interleaved sporadically, with the presence of micritic clasts of 0.1 mm (Figure 6D).

Porosity ranges between 16% and 32%. Pores can be classified as intergranular and cavern type (Choquette & Pray, 1970).

*Non‐laminated zones*: These zones are characterized by the absence of lamination. Only the presence of micrite is observed with micritic clasts, giving rise to mudstone/wackestone textures (Dunham, 1962) (Figure 6E,F). Micritic clasts have rounded morphologies and diameters that are less than 0.25 mm. These non‐laminated zones are interleaved through the internal structure of the microbialite with the laminated areas. Porosity occupies between 18% and 25% of the surface of thin cuts. Pores type can be classified as cavern and intergranular (Choquette & Pray, 1970) (Figure 6E,F).

### Microstructures of microbialitic buildup MIII

In MIII, microbialites forms a continuous biostrome up to 9 cm thick (Table 1). Unlike microbialitic buildups MI and MII, the internal structure of MIII has not been developed around a nucleus and its totally laminated (Figure 3D,H). Its microstructure is composed by the intercalation of continuous and sinuous laminae of micritic and sparitic composition (Figure 3H).

**FIGURE 7:**
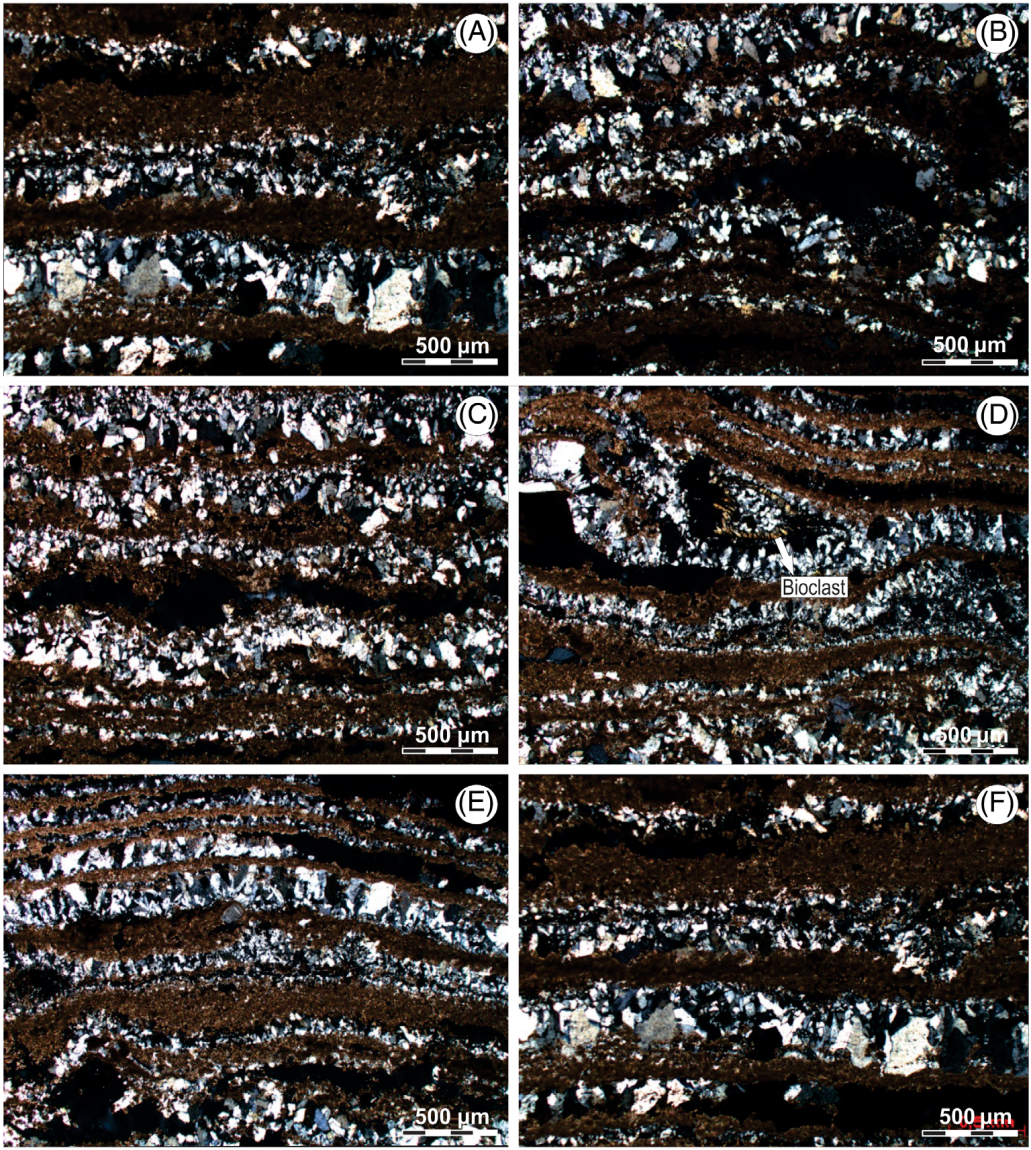
Petrographic microscope images with crosspolars: (A–C, E, F) MIII microstructure, with alternation of micritic and sparite. (D) MIII microstructure, with alternation of micritic and sparite with crystallization inside of a bioclast.

The micritic laminae vary from 0.1 to 0.4 mm and present slightly undulations. Inside, micritic clasts and crystalline material can be identified (Figure 7). Micritic clasts show anhedral rounded morphologies with diameters of up to 0.25 mm, while crystalline clasts show subhedral rounded morphologies and diameters of up to 0.30 mm.

Sparitic laminae reach 0.7 mm thick, presenting anhedral and subhedral crystals up to 0.5 mm (Figure 7F). In some cases, bioclasts can also be observed (possibly ostracod type, due to the shape of the valve) with diameters up to 0.4 mm and with sparitic crystallization inside (Figure 7D).

The x‐ray diffractometry reveals the content of gypsum of 85%, in addition to the presence of calcite (micritic/sparitic) around 14% (Figure 4C). Porosity is approximately 20% of the cut surface, and it can be classified as fenestral, intragranular, intercrystal and intergranular (Choquette & Pray, 1970) (Figure 7).

## DISCUSSION

As it is been mentions by many authors (Dupraz et al., 2006; Mercedes‐Martin et al., 2014; Suosaari et al., 2016), the internal structure of microbialites (in all scales) is the result of the interaction between extrinsic (environmental parameters) and intrinsic factors (microbialite‐producing microorganisms) during their growth. Microstructure is not the exception, and the microbial construction blocks (as individual building units) that forms these organo‐sedimentary structures vary according the environmental conditions (water energy, sedimentation rate, depth, salinity, pH, dissolved oxygen, among others) (Logan, 1961; Suosaari et al., 2019; Wright & Barnett, 2015) and the metabolic activity of the producing microorganisms (Allwood et al., 2009).

### Microstructure interpretation

#### MI and MII

In both MI and MII oncolites, growth starts from a micritic/microsparitic core, which was colonized by benthic microbial communities. This is commonly observed in several Holocene systems of the Puna (Gomez et al., 2014; Villafañe, Lencina, et al., 2021).

The continuous lamination zones show a microstructure formed by biologically induced in situ mineral precipitation (Reid et al., 2000). Alternation between micritic and microsparitic laminae could be attributed to variations in the nucleation/growth relation of the crystals (Dupraz et al., 2009; Riding, 2000). This variations can be due to different factors such as the precipitation rate, fluctuations in the carbonate and calcium concentration, organic activity and even seasonal variations (Dobberschütz et al., 2018; Giuffre et al., 2013; Hu et al., 2012; Li & Jun, 2019).

Both c*lotted laminated zones* in oncolites of MI and *dome laminated zones* in oncolites of MII present the same mineral composition than the c*ontinuous laminated zones* at same level. This suggests that in situ mineral precipitation with microbial influence was the dominant formation process in these oncolites (Gomez et al., 2014; Spadafora et al., 2010; Villafañe, Cónsole‐Gonella, et al., 2021).

‘Clotted’ texture is usually generated because crystalline growth has as a centre of nucleation cyanobacteria (possibly coccoid type) and its metabolic activity might lead the calcium carbonate precipitation in this type of structure (Arenas & Pomar, 2010; Kennard & James, 1986; Monty, 1976; Spadafora et al., 2010). In addition to this, an early diagenetic process could help to preserve them (Spadafora et al., 2010).

In a restricted environment such as Turquesa Lake, where the hydric crisis causes the water level to vary sporadically, domed microstructures could respond to episodes of subaerial exposure generating a retraction of the mats by desiccation (Logan, 1961; Mata et al., 2012). However, we cannot rule out the influence of biotic factors (Von Der Borch et al., 1977).

In oncolites, *non‐laminated zones* can be explained by different factors: (i) Lack of conservation/destruction of the lamination (Reid et al., 2003), (ii) the physical and chemical conditions were not appropriated for the mat growth (Riding, 2008), or (iii) the microbial community that was present during this period does not construct laminated structures (Playford et al., 2013; Suosaari et al., 2016). The possibility that the lamination was destructed is discarded, because during its formation the environment was restricted, of low energy and there is no evidence of metazoans for this microbialitic buildup (Valero‐Garcés et al., 2001). Although petrographic studies cannot suggest variations in the taxa of the microorganisms producing microbialites, this zone has the same mineral composition as the previous ones. For this reason, it is sensible to consider that this microstructural variation responds more to physical factors (such as changes in water level) than to chemical–biological ones (Dupraz et al., 2009; Reid et al., 2000; Riding, 2000).

#### MIII

With a microstructure that is totally composed by an alternation of micritic and sparitic laminae, microbialites of MIII presents remarkable differences with MI and MII. The alternation between micritic and sparitic laminae can be explained by the overlap of in vivo and *post‐mortem* calcareous layers in the microbialite (Kaźmierczak et al., 2015). Micritic laminae is originated as a product of the in situ mineral precipitation with microbial influence (Reid et al., 2000). Gomez et al. (2014) suggest that in Laguna Negra oncolites, micritic laminae take place when the biofilms are better developed and have a stronger control over the microfabric formation. On the other hand, sparitic laminae might be formed by chemical precipitation, in periods of time when crystal growth predominates over the nucleation (Dupraz et al., 2009; Riding, 2000). This texture is associated with a decomposition of the biofilm leading to a nucleation and crystal growth that leave the associated organic material buried (Gomez et al., 2014; Kaźmierczak et al., 2015).

The repeatability of the lamination all through the microstructure of this microbialites indicates a cyclic control (Monty, 1976). This can be influenced by seasonal environmental changes (temperature, water chemistry, nutrient influx), affecting the development of the biofilms, mineral supersaturation, mineral nucleation and a subsequent crystal growth (Dupraz et al., 2006; Gomez et al., 2014; Suosaari et al., 2016).

### Microbialites and environmental crises: Testing analogues from geological records

Microbialites have notably caught the attention of the scientific community for its extensive evidence in the geological record after massive extinctions (Chen et al., 2011; Mata & Bottjer, 2012; Wu et al., 2017; Yang et al., 2011) and important climate changing events (Le Ber et al., 2013; Lipar et al., 2017; Pollier et al., 2021). Therefore, these structures are known as opportunistic and disaster‐related forms in stressed environments (Ezaki et al., 2008), also known as ‘unusual facies’ or ‘disaster form’ (see Duan et al., 2018).

The capacity of resilience of microorganism producing this organo‐sedimentary structures is a key subject to understand the early evolution of life in our planet (Krumbein et al., 2003; Noffke et al., 2006; Webb & Kamber, 2011), and probably the success of its adaptation is due to the complex of the microbial communities acting in the formation of this structures and its big spectrum of metabolic functions (Baud et al., 1997; Breitbart et al., 2009; Iniesto et al., 2021).

Microbialites developed in alkaline lakes under parameters such as high salinity, absence of herbivorous, and eukaryotes bioturbators, hydrothermal influence, among others are considered good textural analogues of Precambrian systems (Popall et al., 2020). If we also take in to consideration that the short time period encompassing the microbialitic record of Turquesa Lake (Villafañe, Cónsole‐Gonella, et al., 2021) and the constant environmental variations registered in this water body (Valero‐Garcés et al., 2001), the importance of this structures as disaster forms increases.

The study of microorganism producing microbialites resilience capacity has been the objective of different works, both in fossil and modern lacustrine environments. Lindsay et al. (2019) and Popall et al. (2020) suggest that microbialites can survive to high salinity values and long‐term UV‐C exposure, respectively, due to a quantitative change in the microbial community. Popall et al. (2020) also mention the especial resilience of the specific type of cyanobacteria to UV‐C increases. On the other hand, Falcón et al. (2020) attribute the resilience of these microbial communities to a diversity of microorganism acting together and intertwining their metabolic capabilities to create self‐sustained microbial ecosystems. On the other hand, in lakes with constant changes in the water level, energy and increases in turbidity, Eymard et al. (2019) suggest that the microorganisms producing microbialites can recolonize the new coasts with changes by their functions. All the factors mention previously are also present in Turquesa Lake (long‐term UV exposure, high salinity, changes in the water level), this supports the idea that the microbial communities in the Turquesa Lake had adapted to harsh and changing environmental conditions (as in the examples from other authors). Both in the paleocoasts (MI and MII) and in the current microbialitic build up (MIII), there is evidence of the resilience of these microorganisms through time.

### Environmental evolution model

**FIGURE 8:**
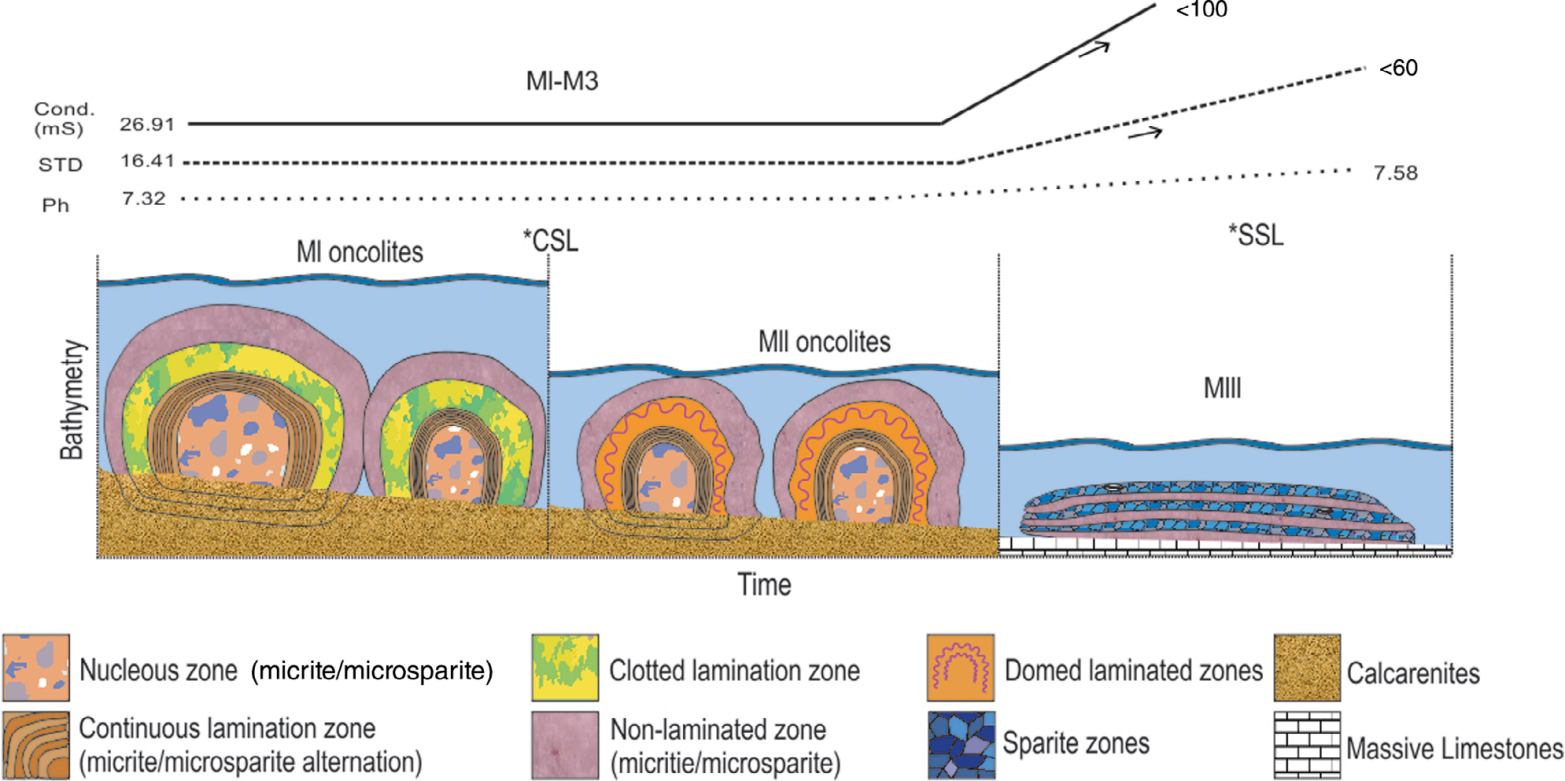
Reconstruction of the variation in the microstructure related with time, bathymetry level and changes on the main parameters (pH, total dissolved solids and conductivity; *Source*: taken from Villafañe, Cónsole‐Gonella, et al., 2021; Villafañe, Lencina, et al., 2021 ) from MI to MIII. *CSL, connected stage between Turquesa Lake and Peinado Lake; *SSL, separation stage of the lakes

As can be seen in its stratigraphic record, microorganisms producing microbialites has colonized the coast of Turquesa Lake in three stages throughout the hydrological crisis. In these stages, extrinsic and intrinsic factors of the environment varied, possibly controlled by the loss of depth and the episodes of connection/disconnection with Peinado Lake (Valero‐Garcés et al., 2001; Villafañe, Cónsole‐Gonella, et al., 2021). This environmental evolution model and the changes in the main parameters could be pictured in Figure 8 of the current work.

*First stage*: Microbialites of MI were formed in a first moment of stability during the hydrological crisis of Turquesa Lake. These ones are developed in a restricted lower intertidal/shallow subtidal environment, connected in a continuous or sporadic way with Peinado Lake. For this mixture of waters, during this stage Turquesa Lake would present similar chemical parameters than those currently observed in Peinado Lake (Valero‐Garcés et al., 2001; Villafañe, Cónsole‐Gonella, et al., 2021).

Around the nucleus, the oncolites microstructure change between c*ontinuous laminated zones*, c*lotted laminated zones*, *dome laminated zones* and *non‐laminated zones*. These changes suggest that during the growth of this microbialitic buildup the environmental conditions were fluctuating. Environmental variations can be explained by episodes of higher/less aridity that would control the connection/disconnection episodes of the lake. Therefore, these sporadic events could affect both chemical composition of the water and quantitative composition of its microbial colonies (Bąbel, 2004).

The precipitation of micritic/microsparitic occurs under subaquatic conditions in which the microbial activity is favoured by the water mix. Mg and Ca are triggered by hydrothermal sources contributed by Peinado lake connection, while carbonate precipitation is triggered by a progressive evaporation and a CO_2_ degassing due to the hydrological crisis of the lake (Beeler et al., 2020; Gomez et al., 2014; Villafañe, Cónsole‐Gonella, et al., 2021). When the system starts to lose depth again, leaving the microbialites exposed to subaerial conditions the growth is interrupted.

*Second stage*: In a second stage, with a new moment of stability during the hydrological crisis, microbialites of MII start to develop. Based on the similarities observed with MI, both in external and internal morphology's (mainly in its microstructure), we can suggest that both had been formed in different stages of water level stability, but under very similar intrinsic and extrinsic conditions (Chen et al., 2011; Wu et al., 2017). Like in MI, MII microbialites grew up in an intertidal/shallow subtidal environment with low hydrodynamic energy.

As in MI microstructure studies suggest that MII presents dynamic environmental variations during its formation. These variations are also due to the episodes of higher/less aridity that control connection/disconnection of the lakes (Solari et al., 2010; Benson and Palliet, 2002). However, textural and mineralogical similarities in the construction blocks of both microbialitic buildups, indicates that environmental conditions during MII formation, were similar to the ones in MI (Chen et al., 2011; Eymard et al., 2019; Wu et al., 2017). This allows us to suggest that despite the loss of depth, the episodes of connection/disconnection between Turquesa and Peinado lakes continue during this stage.

Under the hydric crisis and with the variations in aridity mentioned earlier, it is possible that during the development of the MII microbialitic buildup the microbialites were exposed to sporadic subaerial conditions. This could cause desiccation and cracking of the mats, explaining the passages with *dome laminated zones* (Logan, 1961; Mata et al., 2012). Added to this, an increase in the dissolved solids is generated by the negative hydrological balance, evidenced by the sparitic precipitation in the poral space (Gomez et al., 2014). As the loss of depth continues, microbialites in MII remain totally expose to subaerial conditions and its growth is interrupted.

*Third stage*: In a last stage, marked by a new moment of environmental stability, microbialites of MIII starts to develop. Microbialites at this buildup are currently underwater in an intertidal position. Unlike MI and MII, the microstructure in MIII remains homogeneous, suggesting that extrinsic and intrinsic factors during its formation stayed stable (Dupraz et al., 2006; Mercedes‐Martin et al., 2014).

Nonetheless, an important textural variation is observed compared to the other buildups, given by the sparitic contents. These changes could be explained by a definitive disconnection of Turquesa Lake with Peinado Lake interrupting the hydrothermal supply and increasing the evaporation and water saturation. With this definitive isolation of Turquesa Lake, the chemical conditions would have been modified and MIII began its development in waters with a higher degree of saturation than MI and MII. While these variations can modify quantitative or qualitative the composition of the microbial communities (Baud et al., 1997; Chen et al., 2011), is possible that the complex of the microbial communities allows them to develop in a big spectrum of metabolic functions to quickly proliferate under fluctuating physical and chemical conditions, modifying the construction block and even the forming mechanism if needed (Baud et al., 1997; Sheehan & Harris, 2004).

## CONCLUSIONS

The microstructures of the microbialitic buildups of Turquesa Lake clearly reflect the environmental variables to which this body of water was subjected during its development. Based on their study, it is possible to suggest a three‐stage environmental model.

Initially, the coast of Turquesa Lake is colonized by MI‐producing microorganisms, which present micritic to microsparitic textures generated by in situ biologically induced precipitation. Throughout the growth of this microbialitic buildup, environmental conditions were not stable, conditioned by events of greater and lesser aridity associated with episodes of connection and disconnection with the Peinado Lake. This is clearly expressed in the microstructural variations of these oncolites.

After an evident decrease in the depth of the lake, the MII microbialitic buildup producing microorganisms colonize the new shoreline. The microstructural, mineralogical and textural similarities with MI suggest similar environmental conditions to the ones occurred during the formation of this microbialitic buildup. Variations in the aridity of the system that have had repercussions in episodes of connection and disconnection with the Peinado Lake are factors that are repeated during the formation of MII.

Finally, and after a new bathymetric descent, the MIII structure grow immediately below the air–water interface. Although the construction mechanism continues to be in situ biologically induced carbonate precipitation, important microstructural and textural changes are observed. The oncoid morphology of the previous microbialitic buildups is lost and a platform shape structure with parallel micritic–sparitic lamination is observed. The homogeneity in the microstructure of this buildup responds to more stable environmental conditions throughout its development. While the change in the building block supports a chemical variation in the environment due to the total isolation from Peinado Lake. As concluding remark, microbialite structures from the Argentinean Puna, can be proposed as an interesting modern analogue on changes and adaptations of these systems along the geological record. Based on the results of this work microbialites have demonstrated to be resilient structures, which after abrupt environmental changes can adapt and recolonize the environment in short term.

## CONFLICT OF INTEREST

The author declares that there is no conflict of interest that could be perceived as prejudicing the impartiality of the research reported.

## Data Availability

Data available in article supplementary material.
